# Supplementary material for: Structural transition and re-emergence of iron's total electron spin in (Mg,Fe)O at ultrahigh pressure
Source: Nat Commun. 2022 May 19;13:2780. doi: 10.1038/s41467-022-30100-5 (PMC9120148; doi:10.1038/s41467-022-30100-5)
Supplement: Supplementary file 1 — Supplementary Information for “Structural transition and re-emergence of iron’s total electron spin in (Mg,Fe)O at ultrahigh pressure” [file 41467_2022_30100_MOESM1_ESM.pdf]

Supplementary Information for "Structural transition and re-emergence of iron's total electron spin in (Mg,Fe)O at ultrahigh pressure"

Han Hsu[1,*] and Koichiro Umemoto[2]

[1]*Department of Physics, National Central University, Taoyuan City 32001, Taiwan*

[2]*Earth-Life Science Institute, Tokyo Institute of Technology, Tokyo, 152-8550, Japan*

(Dated: March 31, 2022)

* Corresponding author: hanhsu@ncu.edu.tw

Supplementary Note 1. B1 (Mg,Fe)O

In B1 ($Mg_{1-x}Fe_x$)O, Fe substitutes Mg in the 6-coordinate octahedral site (Supplementary Fig. 1a). In such a crystal field, the Fe t_{2g} orbitals have lower energy than the e_g orbitals, and the orbital configurations of the high-spin (HS) and low-spin (LS) states are $t_{2g}^4 e_g^2$ and $t_{2g}^6 e_g^0$, respectively (Supplementary Fig. 1b). Using the local density approximation + self-consistent Hubbard U (LDA+U_{sc}) method, the density of states (DOS) of B1 LS ($Mg_{0.75}Fe_{0.25}$)O at volume $V = 8.829$ Å^3/f.u. (pressure $P = 0.559$ TPa) is computed, as shown in Supplementary Fig. 1c. A gap is opened between the t_{2g} and e_g bands, indicating that B1 LS ($Mg_{0.75}Fe_{0.25}$)O is insulating.

Supplementary Note 2. EQUATION OF STATE

To obtain the equations of state (EoS) of B1 and (r)B2 ($Mg_{1-x}Fe_x$)O, energies (E) of these structural phases at various volumes are computed and then fitted with the third-order Birch–Murnaghan equation of state (3rd BM EoS). For $x = 0.125$ and 0.25, the computed energy (via LDA+U_{sc}) and the fitted $E(V)$ curves of B1 LS, (r)B2 intermediate-spin (IS), and (r)B2 LS states are shown in Supplementary Fig. 2. For each composition, the reference energy (0 eV) is set at the equilibrium energy of the B1 LS state.

Supplementary Note 3. B1–B2 TRANSITION OF MgO

As described in the main text, various experiments and calculations have indicated that the B1–B2 structural transition of Fe-free MgO occurs at ∼0.5 TPa. In our LDA calculation, the B1–B2 transition occurs at 0.535 TPa (Supplementary Fig. 3), in agreement with previous works.

Supplementary Note 4. DFT FUNCTIONALS AND HUBBARD U PARAMETERS

Within the density functional theory + Hubbard U (DFT+U) framework, the choice of the exchange-correlation functional [LDA or generalized-gradient approximation (GGA)] and the Hubbard U parameter directly affects the calculation results, especially (1) the most energetically favorable spin state at a given pressure, and (2) the spin-transition pressure.

While LDA+U_{sc} has been established as a reliable approach to study Fe-bearing minerals at high pressure, we still perform extensive test calculations using various choices of DFT+U to examine the robustness of the LDA+U_{sc} results shown in the main text (Fig. 6), as described below.

For transition-metal compounds, GGA+U with a constant U is a widely adopted method. In Supplementary Fig. 4, GGA+U calculations for $(Mg_{1-x}Fe_x)O$ with U = 6, 7, and 8 eV are shown. In these calculations, the obtained sequence of the structural and spin transitions are the same as the LDA+U_{sc} results (Fig. 6). For $x = 0.125$ (Supplementary Fig. 4a–c), a transition from the B1 LS to the rB2 IS state occurs at $\sim$ 0.63 TPa, followed by an IS–LS transition in the rB2 phase. For $x = 0.25$ (Supplementary Fig. 4d–f), a transition from the B1 LS to the B2 IS state occurs at $\sim$ 0.54 TPa, followed by an IS–LS transition in the B2 phase. For both compositions ($x = 0.125$ and 0.25), the predicted B1–rB2 and B1–B2 transition pressures are barely affected by the U parameter. In contrast, the predicted IS–LS spin-transition pressure in (r)B2 $(Mg_{1-x}Fe_x)O$ significantly increases with U. As U increases from 6 to 8 eV, the IS–LS transition pressure increases from 1.160 to 1.247 TPa for $x = 0.125$ (Supplementary Fig. 4a–c) and from 0.801 to 0.957 TPa for $x = 0.25$ (Supplementary Fig. 4d–f), namely, as U increases by 2 eV, the predicted IS–LS transition pressure increases by $\sim$ 0.1 TPa (100 GPa). In short, all these GGA+U calculations show highly similar results with each other and with LDA+U_{sc} (Fig. 6). The main difference between these results is the IS–LS transition pressure in the (r)B2 phase. Overall, the results obtained using GGA+U with U = 7 eV (Supplementary Fig. 4b, e) are in best agreement with LDA+U_{sc} (Fig. 6).

In Supplementary Fig. 5, LDA+U results with U = 10 and 14 eV are shown. Within LDA+U, an exceptionally large U (> 10 eV) is necessary to stabilize the rB2 IS state for $x = 0.125$. When U = 10 eV (Supplementary Fig. 5a), the enthalpy crossing of the rB2 LS and rB2 IS states occurs at 0.516 TPa, lower than the B1–rB2 transition pressure (0.648 TPa), namely, a transition from the B1 LS to the rB2 LS state occurs at 0.648 TPa without going through the rB2 IS state. When U increases to 14 eV (Supplementary Fig. 5b), rB2 IS becomes the most favorable state in the pressure region of 0.638–0.821 TPa, and the sequence of the transitions becomes B1 LS $\rightarrow$ rB2 IS $\rightarrow$ rB2 LS, same as the LDA+U_{sc} (Fig. 6) and GGA+U results (Supplementary Fig. 4). For $x = 0.25$ (Supplementary Fig. 5c,

d), the sequence of the transition remains B1 LS $\rightarrow$ B2 IS $\rightarrow$ B2 LS, regardless of the choice of U. As indicated by Supplementary Fig. 5, the predicted B1–(r)B2 structural transition pressure are ~ 0.64 and ~ 0.54 TPa for $x = 0.125$ and 0.25, respectively, barely affected by the choice of U, and nearly the same as the LDA+U_{sc} (Fig. 6) and GGA+U results (Supplementary Fig. 4). In contrast, the predicted IS–LS transition pressure in (r)B2 $(Mg_{1-x}Fe_x)O$ significantly increases with U: from 0.516 to 0.821 TPa for $x = 0.125$ (Supplementary Fig. 5a, b) and from 0.934 to 1.199 TPa for $x = 0.25$ (Supplementary Fig. 5c, d), namely, as U increases by 4 eV, the predicted IS–LS transition pressure increases by > 0.2 TPa (200 GPa).

Supplementary Note 5. THERMODYNAMIC MODEL

In this section, we detail the thermodynamic model for spin transition at nonzero temperature (T). This model has also been described in Refs. 19, 25, 29, 51, and 52 cited in the main text. At $T \neq 0$, spin transition of B2 $(Mg_{1-x}Fe_x)O$ goes through a mixed-spin (MS) phase/state, in which all spin states coexist. The fraction of spin state i (i = HS, IS, or LS) in the MS phase is written as $n_i(P,T)$. Here the MS phase is considered a solid solution consisting of all spin states, and its Gibbs free energy is written as

$$G(P,T) = \sum_i n_i(P,T)G_i(P,T) - TS^{mix}, \tag{1}$$

where $G_i(P,T)$ is the Gibbs free energy of spin state i, and S^{mix} is the mixing entropy of the solid solution, given by

$$S^{mix} = -k_B x \sum_i n_i \ln n_i. \tag{2}$$

The Gibbs free energy G_i of spin state i is contributed by several factors, written as

$$G_i(P,T) = G_i^{vib+stat} + G_i^{mag}. \tag{3}$$

In Supplementary Eq. (3), the second term is magnetic contribution, derived from the magnetic entropy

$$G_i^{mag} = -k_B T x \ln(2S_i^{el} + 1), \tag{4}$$

where S_i^{el} is the total electron spin of state i. For Fe^{2+}, $S_{HS}^{el} = 2$, $S_{IS}^{el} = 1$, and $S_{LS}^{el} = 0$. The first term, $G_i^{vib+stat}$, contains static and vibrational contributions. If the vibrational

contribution is disregarded, $G_i^{vib+stat}$ is reduced to the static enthalpy H_i of spin state i, and the Gibbs free energy is written as

$$G_i(P,T) = H_i - k_B T x \ln(2S_i^{el} + 1). \tag{5}$$

For B2 $(Mg_{1-x}Fe_x)O$, only the IS and LS states are relevant (see the main text), namely, $n_{LS} + n_{IS} = 1$. For convenience, we write $n \equiv n_{LS}$ and $n_{IS} = 1 - n$. Using this notation, the Gibbs free energy and mixing entropy in Supplementary Eqs. (1) and (2) are rewritten as

$$G(n,P,T) = nG_{LS} + (1-n)G_{IS} - TS^{mix}, \tag{6}$$

$$S^{mix} = -k_B x[n \ln n + (1-n)\ln(1-n)]. \tag{7}$$

At equilibrium, the Gibbs free energy is minimized, namely, $(\partial G/\partial n)_{P,T} = 0$. By taking the derivatives of Supplementary Eqs. (6) and (7) with respect to n, we obtain

$$n = \frac{1}{1 + \exp(\Delta G_{LS}/k_B T x)}, \tag{8}$$

where $\Delta G_{LS} \equiv G_{LS} - G_{IS}$. Without the inclusion of vibrational free energy, based on Eq. (5), the LS fraction n in Supplementary Eq. (8) is written as

$$n = \frac{1}{1 + (2S_{IS}^{el} + 1)\exp(\Delta H_{LS}/k_B T x)}, \tag{9}$$

where $\Delta H_{LS} \equiv H_{LS} - H_{IS}$, and $2S_{IS}^{el} + 1 = 3$. With the LS fraction (n) obtained, the Gibbs free energy of the MS phase can also be obtained via Supplementary Eqs. (6) and (7), and the equation of state (EoS) of the MS phase can be derived accordingly

$$V = \left(\frac{\partial G}{\partial P}\right)_T = nV_{LS} + (1-n)V_{IS}, \tag{10}$$

where V_{LS} and V_{IS} are the EoS of the LS and IS states, respectively. In the main text, the results shown in Fig. 7 are computed based on Supplementary Eqs.(9) and (10).

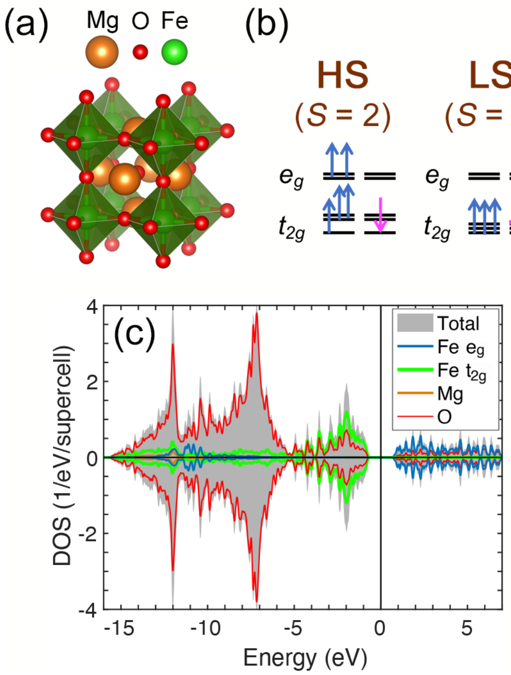

Supplementary Fig. 1. Insulating B1 $(\mathrm{Mg}_{1-x}\mathrm{Fe}_x)\mathrm{O}$ at high pressure. **a** Atomic structure of B1 $(\mathrm{Mg}_{0.75}\mathrm{Fe}_{0.25})\mathrm{O}$; **b** Fe 3$d$ orbital occupations of B1 HS and LS states; **c** total and projected DOS of B1 LS $(\mathrm{Mg}_{0.75}\mathrm{Fe}_{0.25})\mathrm{O}$ at $V = 8.829$ Å^3/f.u. ($P = 0.559$ TPa), with the Fermi level set as the reference (0 eV). Here, the DOS is computed using the LDA+U_{sc} method.

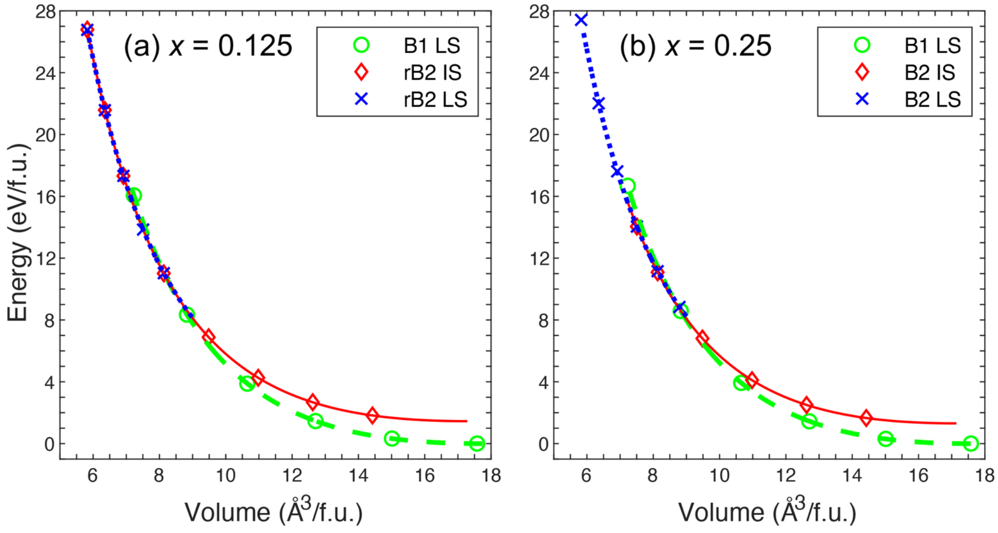

Supplementary Fig. 2. Energy of B1 and (r)B2 $(Mg_{1-x}Fe_x)O$ in various spin states and volumes. Direct LDA+U_{sc} calculation results (symbols) are fitted with the 3rd BM EoS (lines). The equilibrium energy of the B1 LS state is used as the reference (0 eV). **a** $x = 0.125$; **b** $x = 0.25$.

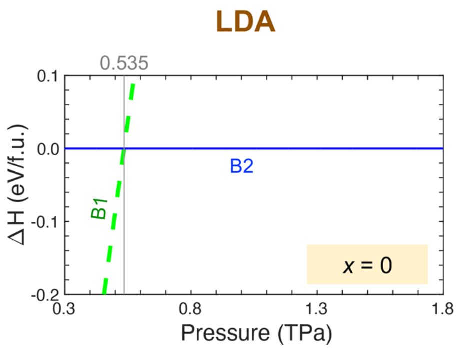

Supplementary Fig. 3. Relative enthalpy of Fe-free B1 MgO ($x = 0$) with respect to the B2 phase. Here the LDA results are shown. The vertical line and the number above indicate the enthalpy crossing and transition pressure, respectively.

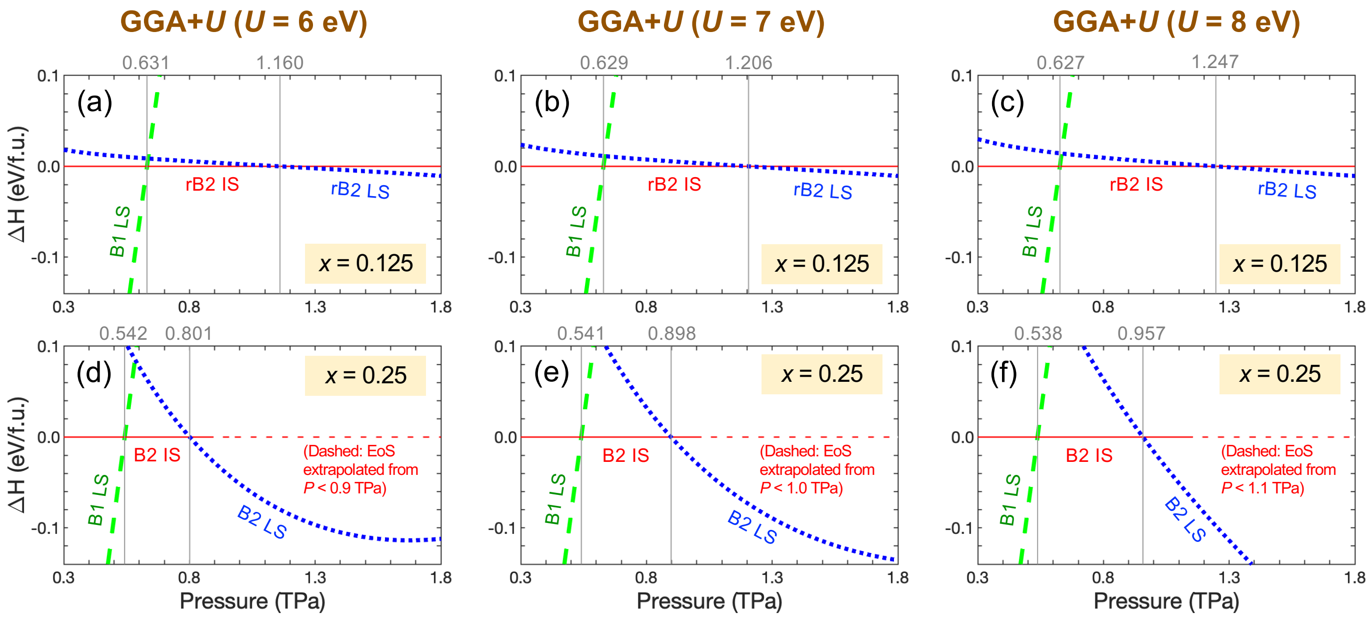

Supplementary Fig. 4. Relative enthalpies of $(Mg_{1-x}Fe_x)O$ in various structural phases and spin states determined using GGA+U with $U = 6$ (**a, d**), 7 (**b, e**), and 8 (**c, f**) eV. The reference states are rB2 IS for $x = 0.125$ (**a–c**) and B2 IS for $x = 0.25$ (**d–f**). The vertical lines and the numbers above indicate the enthalpy crossings and transition pressures, respectively.

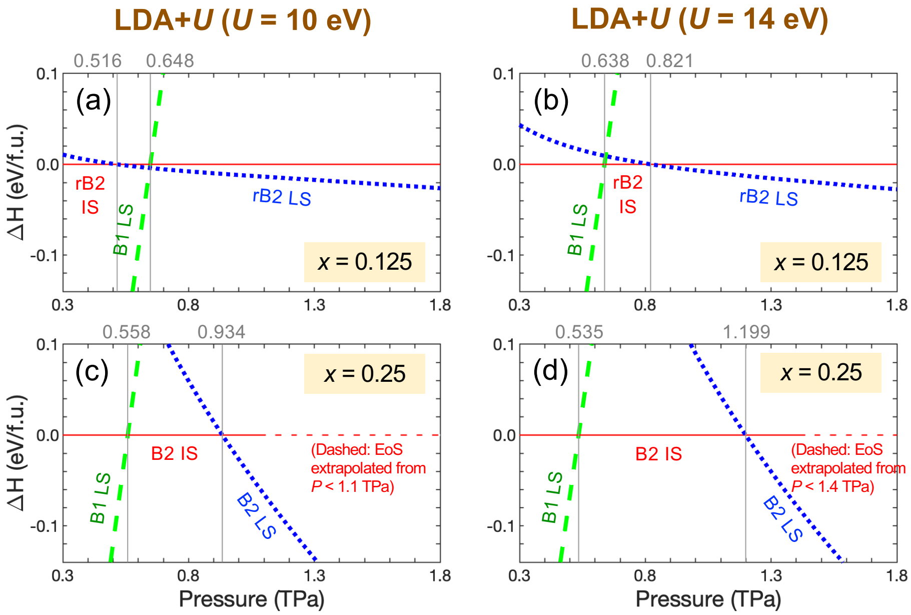

Supplementary Fig. 5. Relative enthalpies of $(Mg_{1-x}Fe_x)O$ in various structural phases and spin states determined using LDA+U with U = 10 (**a, c**) and 14 (**b, d**) eV. The reference states are rB2 IS for $x = 0.125$ (**a, b**) and B2 IS for $x = 0.25$ (**c, d**). The vertical lines and the numbers above indicate the enthalpy crossings and transition pressures, respectively.
